# Identification of the transcripts associated with spontaneous HCV clearance in individuals co-infected with HIV and HCV

**DOI:** 10.1186/s12879-016-2044-7

**Published:** 2016-11-22

**Authors:** Yue Chen, Chengli Shen, Debjani Guha, Ming Ding, Scott Kulich, Aiymkul Ashimkhanova, Charles Rinaldo, Eric Seaberg, Joseph B. Margolick, Valentina Stosor, Otoniel Martínez-Maza, Phalguni Gupta

**Affiliations:** 1 Department of Infectious Diseases and Microbiology, Graduate School of Public Health, University of Pittsburgh, 2138 Parran Hall, 130 DeSoto Street, Pittsburgh, Pa 15261 USA; 2 Department of Pathology, VA Hospital, Pittsburgh, Pa USA; 3 Department of Molecular Microbiology and Immunology, Bloomberg School of Public Health, Johns Hopkins University, Baltimore, MD USA; 4 Division of Infectious Diseases, School of Medicine, Northwestern University, Chicago, IL USA; 5 Department of Epidemiology, UCLA Fielding School of Public Health, and Departments of Obstetrics & Gynecology and Microbiology, Immunology & Molecular Genetics, David Geffen School of Medicine at UCLA, Los Angeles, CA USA

**Keywords:** HIV/HCV coinfection, Clearance of HCV infection, Chronic HCV infection, Plasma RNA sequencing, Sequence analysis

## Abstract

**Background:**

Infection with human immunodeficiency virus (HIV) influences the outcome and natural disease progression of hepatitis C virus (HCV) infection. While the majority of HCV mono-infected and HCV/HIV co-infected subjects develop chronic HCV infection, 20–46% of mono- and co-infected subjects spontaneously clear HCV infection. The mechanism underlying viral clearance is not clearly understood. Analysis of differential cellular gene expression (mRNA) between HIV-infected patients with persistent HCV infection or spontaneous clearance could provide a unique opportunity to decipher the mechanism of HCV clearance.

**Methods:**

Plasma RNA from HIV/HCV co-infected subjects who cleared HCV and those who remained chronically infected with HCV was sequenced using Ion Torrent technology. The sequencing results were analyzed to identify transcripts that are associated with HCV clearance by measuring differential gene expression in HIV/HCV co-infected subjects who cleared HCV and those who remained chronically infected with HCV.

**Results:**

We have identified plasma mRNA, the levels of which are significantly elevated (at least 5 fold, False Discovery Rate (FDR) <0.05) before HCV infection in subjects who cleared HCV compared to those who remained chronically infected. Upon further analysis of these differentially expressed genes, before and after HCV infection, we found that before HCV infection 12 genes were uniquely upregulated in the clearance group compared to the chronically infected group. Importantly, a number of these 12 genes and their upstream regulators (such as CCL3, IL17D, LBP, SOCS3, NFKBIL1, IRF) are associated with innate immune response functions.

**Conclusions:**

These results suggest that subjects who spontaneously clear HCV may express these unique genes associated with innate immune functions.

## Background

Hepatitis C virus (HCV) infection is the leading cause of hepatocellular carcinoma in the United States [1]. Due to similar transmission routes, approximately one third of HIV-infected individuals are co-infected with HCV [2–4]. In HCV mono infection, nearly 70–80% of infected individuals develop chronic infection and liver cirrhosis, some of whom develop hepatocellular carcinoma, while 20–30% of infected individuals spontaneously clear the viral infection within one year of infection [5]. However, in HIV/HCV co-infected individuals, infection with HIV is associated with a decrease in the rate of spontaneous HCV clearance, exacerbates the natural course of HCV infection, and accelerates the development of HCC [6–10]. Currently, the mechanism of spontaneous HCV clearance and how HIV might affect this are not clear. Certain immunological components have been shown to be involved in HCV clearance [11–13]. An HCV-specific T-cell response in the liver has also been reported to be associated with HCV clearance. Various factors, such as gene polymorphisms in interleukin 28B (*IL28B)* and *IP-10* have been implicated in mediating host susceptibility/resistance to HCV infection and disease progression [14–17]. In HCV-infected chimpanzees, interleukin binding factor 3 (ILF3) and cytotoxic granule associated cRNA binding protein (TIA1), which are associated with robust T-cell responses, were highly induced in animals who cleared the virus [18]. However, these represent only a few of the many factors that are potentially involved in HCV clearance.

Differential cellular gene expression in HIV-infected subjects has been shown to be associated with HIV disease progression [19]. Therefore, analysis of differential cellular gene expression between HIV infected patients with persistent HCV infection and those with spontaneous clearance of this virus could provide a unique opportunity to decipher the mechanism of HCV clearance in HIV/HCV co-infected subjects.

Pathogenic changes occurring in any tissues or organs are expected to leave footprints in the blood. Consequently, measurement of certain target RNA in the plasma has been explored or used for diagnosis of a number of diseases and cancers [20–24]. We hypothesize that the cellular (immunological and non-immunological) factors that are responsible for spontaneous HCV clearance leave footprints in the plasma, leading to differential plasma RNA expression profiles between those who spontaneously clear HCV infection and chronically infected individuals, with or without HIV co-infection. It is presumed that differential gene expression in plasma between those who cleared HCV and those who remained chronically infected will provide clues to the mechanism of HCV clearance. Due to the low quantity of RNA in blood plasma, currently there is no convenient way to characterize plasma RNA profiles. Recent advancement of next-generation sequencing technologies has made it possible for unbiased and comprehensive analysis of the gene expression from both cells and tissues. We have recently modified the NGS technology in Ion Proton platform and characterized RNA profiles in plasma.

In this study we identified plasma mRNA, the levels of which are significantly increased (at least 5 fold, FDR <0.05) in the HCV clearance group compared to chronically HCV infected patients. Upon further analysis of these differentially expressed genes, we have identified 12 genes that are upregulated only in clearance group before HCV infection. Moreover, some of these 12 genes and their upstream regulators are associated with innate immune functions.

## Methods

### Patients and samples

Frozen plasma samples were obtained from 13 HCV seroconverters who were infected with HIV for more than 10 years before HCV seroconversion in the Multicenter AIDS Cohort Study (MACS) [25]. MACS is the first and largest study specifically created to examine the natural history of AIDS. MACS participants are seen every six months and at each visit, plasma is taken for storage at -80 °C. HCV seroconversion is defined by the HCV antibody switching from negative to positive in plasma and the plasma HCV antibody positivity were persistent for all subsequent visits of the individuals. Furthermore, HCV seroconversion visits were confirmed by reverse transcriptase polymerase chain reaction specific for detecting HCV RNA. Spontaneous HCV clearance is defined as plasma HCV RNA were never detected or detected only one to two times around the time of seroconversion. Chronic HCV infection was defined as plasma HCV RNA being persistently detected for more than five-ten years. From five subjects with spontaneous HCV clearance and eight subjects with chronic HCV infection, we examined their plasma samples that were obtained immediately before and after HCV infection (MACS visits are six months apart). All HCV infections were reported to have been acquired by sexual transmission. At the time of HCV infection, all subjects were naïve to antiretroviral therapy and anti-HCV treatment.

### Nucleic acid extraction, cDNA library construction and sequencing

Total nucleic acids were extracted from l ml of frozen cryopreserved plasma using an automated NucliSens EasyMag nucleic acid extraction machine (bioMérieux, Durham, NC) followed by removal of DNA from the nucleic extract with Qiagen AllPrep DNA/RNA mini kit and ribosomal RNA by a Low Input RiboMinus System (Life Technologies). The cDNA Library was then constructed using an Ion Torrent Total RNA-Seq Kit (Life Technologies) for whole transcriptome libraries and Barcodes 1 through 8 from an Ion Xpress 1–16 barcoding kit were used (Life Technologies) for each individual sample. cDNA libraries were quantified by qPCR using an Ion Library Quantitation Kit (Life Technologies) to determine a suitable template dilution factor for subsequent emulsion PCR and sequencing.

Four barcoded samples were combined for one sequencing reaction. Template preparation for sequencing was conducted using the OneTouch Ion™ Template Kit in the OneTouch machine (Life Technologies). Ion Torrent sequencing was conducted using the Ion Proton Sequencing Kit (Life Technologies) on an Ion Proton Machine (Life Technologies) using a P1(v2)-chip (Life Technologies). The constructed cDNA was also used for HIV viral load measurement using a quantitative-real time PCR for HIV gag RNA, with a sensitivity of 10 copies/mL [26].

### Sequence analysis

Raw sequencing reads in FastQ format were assessed for quality using CLC Genomics Workbench 7. Reads were accepted based on the length (longer than 25 nucleotides) and number of ambiguous bases (Phred Quality Score higher than 20). Quality trimming was performed. The mean numbers of reads from different groups after trimming ranged from 3.2 million to 8.4 million. The trimmed reads were then mapped to Homo sapiens gene sequences based on Homo sapiens (hg19) mRNA.

Bioconductor edgeR in R package was employed to perform the differential expression analysis. A general linear model was applied on the subjects before and after HCV infection to accommodate the multifactor design of the experiment. The model incorporates the main effect for HCV infection plus interactions with patients and viral clearance, thus allowing us to identify genes differentially expressed in HCV cleared and chronically infected patients before and after HCV infection. To ensure there were sufficient counts for each gene in the test, genes with mean read counts higher than 10 were kept in the analysis. Genes with Benjamini-Hochberg adjusted FDR <0.05 and fold change greater than 1 were considered as significant genes. The significance and function networks of the detected differentially expressed genes were analyzed using Ingenuity Pathway Analysis software. The gene function information was obtained from GeneCards Human Gene Database (www.genecards.org).

## Results

### Characteristics of study participants

A total of 26 plasma samples from the 13 HCV seroconverters obtained within six months before and after HCV infection were analyzed for plasma transcriptome profiles. All the participants were HIV positive for more than 10 years before HCV infection occurred. Four of the five participants who spontaneously cleared HCV infection had the CC variant of the IL28B gene, while the remaining one had the TT variant. In contrast, half of eight participants who became chronically infected by HCV had the CC variant IL28B gene and the remaining participants had the CT variant. For the 5 individuals with spontaneous HCV clearance, HCV loads were undetectable at the first HCV antibody positive visits. In contrast, the eight participants with chronic HCV infection had a median plasma HCV load of 1.6x10^7^ copies/ml at the first HCV RNA positive visit, with values ranging from 1.22 × 10^6^ to 7 × 10^7^ (Table 1). In the HCV clearance group, the HIV viral load was below 40 copies/ml in three of the five patients and > 71,000 copies/ml in two participants both before and after HCV seroconversion. In the chronically HCV infected group, HIV viral load ranged from 360 to 465,000 copies/ml (mean 125,233 copies/ml) (Table 1).

**Table 1:** Clinical characteristics of the study subjects

HCV Clearance Group				
Patient ID	HCV infection	HCV load	HIV load	Polymorphism of IL28B gene
1	-	<43	<40	CC
1	+	<43	<40	
2	-	<43	<40	TT
2	+	<43	<40	
3	-	<43	1,678,080	CC
3	+	<43	3,598,880	
4	-	<43	71,680	CC
4	+	<43	144,480	
5	-	<43	<40	CC
5	+	<43	<40	
HCV Chronic Infection Group				
Patient ID	HCV infection	HCV load	HIV load	Polymorphism of IL28B gene
11	-	<43	129,080	CT
11	+	1.22x10^6^	316,240	
12	-	<43	360	CT
12	+	70x10^6^	50,840	
13	-	<43	81,240	CC
13	+	7.55x10^6^	17,480	
14	-	<43	136,760	CC
14	+	3.93x10^6^	1,040	
15	-	<43	73,840	CC
15	+	13.3x10^6^	21,280	
16	-	<43	465,720	CT
16	+	18.9x10^6^	170,120	
17	-	<43	138,520	CC
17	+	4.8x10^6^	331,560	
18	-	<43	51,400	CT
18	+	7.33x10^6^	18,240	

Minimal detection limit of HIV measurement: 40 copies/ml

Minimal detection limit of HCV measurement: 43 copies/ml

### Plasma mRNA profile of HIV-infected individuals with HCV clearance and those with chronic infection

**Fig. 1:**
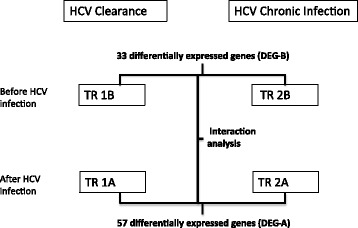
Schematic diagram of the differentially expressed genes from the patients’ plasma samples collected before and after HCV infection. The sequences generated from the patients’ plasma RNA were assessed by CLC Genomics Workbench 7 and statistically analyzed using Bioconductor Edge R

**Fig. 2:**
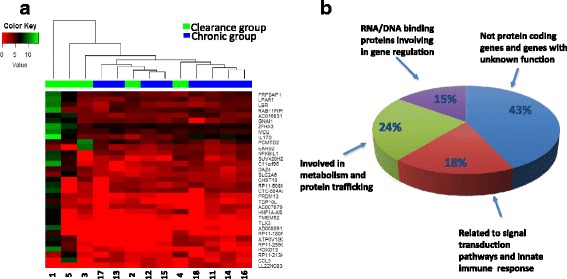
Differentially expressed genes before HCV infection in clearance group compared to chronic group. **a** The heatmap was generated with the statistical differentially expressed genes between clearance group and chronic group before HCV infection. The numbers on X-axis represent individual subjects. Green stands for the clearance group and blue stands for the chronic group. **b** Pie chart illustrates the percentage of the DEGs, which encode proteins involved in different functions

Plasma transcriptome analysis was performed in HCV infected individuals who cleared virus and those who remained chronically infected. A schematic diagram of the approaches for measurement of differentially expressed genes from the patients’ plasma samples collected before and after HCV infection and those cleared HCV and those who remained chronically infected is shown in Fig. 1. A comparison of transcriptomes before HCV infection between the HCV clearance group (TR1B, Fig. 1) and the chronically HCV infected group (TR 2B, Fig. 1) showed that the expression levels of 32 genes were significantly higher (5–563 fold) and expression level of one gene (LL22NC03–63E9.3) was significantly lower in the clearance group (Table 2). To further assess the clustering of subjects within the chronic and clearance groups and across the groups, hierarchical clustering was performed using the differentially expressed genes (Fig. 2a). Four of the eight chronically infected participants clustered together indicating a distinct gene expression pattern whereas the other four samples did not show a similar pattern. On the other hand, three of the five participants in the clearance group clustered indicating similar gene expression and the remaining two subjects had different gene expression. In both clearance and chronic groups, the characteristics of the samples that did not aggregate with the rest of the samples in the respective groups were not different with respect to HIV viral load or *IL28B* polymorphism. This suggests that HIV viral load and *IL28B* polymorphism may not have an independent effect on the pattern of the gene expression.

**Table 2:** DEGs in plasma before HCV infection in clearance group compared to those in chronically infected group

DEGs^a^	Gene function	Fold difference	*P* value	FDR
AD000091.2	Uncharacterized	563.4	2.83E–07	0.001188912
C11orf95	Chromosome 11 open reading frame 95. Diseases associated with C11orf95 include ependymoma and chondroid lipoma.	100.9	5.80E–07	0.001188912
LPAR1	Lysophosphatidic acid receptor 1. Its related pathways are PI3K-Akt signaling pathway and Ras signaling pathway.	20	6.66E–07	0.001188912
LBR	Lamin B receptor. Its related pathways are Metabolism and Metabolism.	20.6	2.85E–06	0.003815476
GNAI1	Guanine nucleotide binding protein (G protein), alpha inhibiting activity polypeptide 1. Its related pathways are GPCR Pathway and GPCR Pathway.	7.6	9.21E–06	0.008563212
HOXD13	Homeobox D13. This gene is related to *sequence-specific DNA binding transcription factor activity and chromatin binding.*	231.4	9.61E–06	0.008563212
EARS2	Glutamyl-tRNA synthetase 2, mitochondrial. Its related pathways are Metabolism and Gene Expression.	22.6	1.17E–05	0.008563212
PRDM13	PR domain containing 13. This gene related to *methyltransferase activity.*	30.4	1.28E–05	0.008563212
RAB11FIP5	RAB11 family interacting protein 5 (class I). Its related pathways are Endocytosis and Delta508-CFTR traffic/ER-to-Golgi in CF.	23	2.52E–05	0.013834245
CTC-504A5.1	Affiliated with the ncRNA class	16.6	2.58E–05	0.013834245
CHST10	Carbohydrate sulfotransferase 10. Its related pathways are Other types of O-glycan biosynthesis and Biological oxidations.	8.9	4.28E–05	0.019890845
PRPSAP1	Phosphoribosyl pyrophosphate synthetase-associated protein 1. This gene is related to *magnesium ion binding* and *enzyme inhibitor activity.*	10	4.46E–05	0.019890845
TLX3	T-cell leukemia homeobox 3. Its related pathways are Transcriptional misregulation in cancer and SIDS Susceptibility Pathways.	103.6	5.11E–05	0.021040208
RP11-259G18.1	Uncharacterized	21.7	7.50E–05	0.024582733
DAZ4	Deleted in azoospermia 1. This gene is related to this gene include *RNA binding* and *nucleotide binding.*	7	7.68E–05	0.024582733
TMEM52	Transmembrane protein 52	50.8	7.68E–05	0.024582733
AC007879.2	Uncharacterized	13.3	7.93E–05	0.024582733
PCMTD2	Protein-L-isoaspartate (D-aspartate) O-methyltransferase domain containing 2. This gene is related to *protein-L-isoaspartate (D-aspartate) O-methyltransferase activity.*	10.4	8.26E–05	0.024582733
HNF1A-AS1	HNF1A antisense RNA 1 affiliated with the antisense RNA class.	13.6	8.86E–05	0.024984166
SUV420H2_1	Uncharacterized	21.7	9.76E–05	0.026140534
ATP6V1G2	ATPase, H+ transporting, lysosomal 13 kDa, V1 subunit G2. A multisubunit enzyme that mediates acidification of intracellular compartments of eukaryotic cells.	27.3	1.06E–04	0.027114541
TCP10L	T-complex 10-like. An important paralog of this gene is TCP10L2.	13.9	1.23E–04	0.028632706
MCU	Mitochondrial calcium uniporter. It is related to *calcium channel activity* and *uniporter activity.*	7.8	1.23E–04	0.028632706
ZFHX3	Zinc finger homeobox 3. Its related pathways are Transcriptional Regulatory Network in Embryonic Stem Cell.	8	1.59E–04	0.035570625
RP11-508 M8.1	Uncharacterized	10.7	1.85E–04	0.039536736
CCL3	Chemokine (C-C motif) ligand 3. Its related pathways are Signaling by GPCR and TGF-Beta Pathway.	31	2.13E–04	0.043901777
LL22NC03-63E9.3	Uncharacterized LOC648691, affiliated with the ncRNA class.	−22.2	2.30E–04	0.045120747
RP11-180P8.3	Uncharacterized	46.1	2.36E–04	0.045120747
RP11-213H15.3	Uncharacterized	11.1	2.56E–04	0.046011494
IL17D	Interleukin 17D. Its related pathways are IL-17 Family Signaling Pathways and IL-17 Family Signaling Pathways.	40	2.58E–04	0.046011494
AC016831.7	Uncharacterized	5.8	2.71E–04	0.04688832
NFKBIL1	Nuclear factor of kappa light polypeptide gene enhancer in B-cells inhibitor-like 1	8	2.98E–04	0.049193252
SLC2A6	Solute carrier family 2, member 6. Its related pathways are PAK Pathway and HIF1Alpha Pathway.	7.9	3.03E–04	0.049193252

^a^ *DEG* Differentially expressed gene

Of the 33 genes that were differentially expressed (DEGs) before HCV infection, 19 DEGs have known biological functions (Gene Cards Human Gene Database (http://www.genecards.org/)): six gene products (18%) (ATP6V1G2, LPAR1, GNAI1, CCL3, IL17D, SLC2A6) are related to signal transduction pathways and innate immune response,. eight gene products (24%) (LBR, EARS2, RAB11FIP5, CHST10, PRPSAP1, PCMTD2, MCU, PRDM13) are involved in metabolism and protein trafficking, and five gene products (15%) (ZFHX3, DAZ4, TLX3, HOXD13, NFKBIL1**)** are RNA/DNA binding proteins involved in gene regulation (Fig. 2b).

**Fig. 3:**
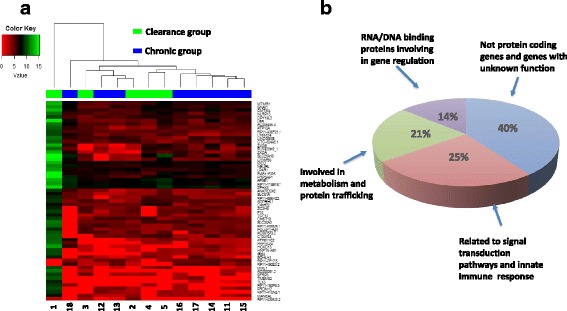
Differentially expressed genes after HCV infection in clearance group compared to chronic group. **a** The heatmap was generated with the statistical differentially expressed genes between clearance group and chronic group after HCV infection. The numbers on X-axis represent individual subjects. Green stands for the clearance group and blue stands for the chronic group. **b** The pie chart illustrates the percentage of the DEGs, which encode proteins involved in different functions

To determine the differential gene expression pattern in response to HCV infection, a similar analysis was performed between transcriptomes in the clearance group (TR1A, Fig. 1) and chronic infection group (TR2A, Fig. 1), after HCV infection (Fig. 1). The expression levels of 56 genes were significantly higher, and that of one gene was significantly lower, in TR1A compared to TR2A (Table 3). Hierarchical clustering of DEGs showed that genes from five patients who had chronic infection clustered together, while no such clustering pattern was observed in the clearance group (Fig. 3a).

**Table 3:** DEGs in plasma after HCV infection in clearance group compared to those in chronically infected group

DEGs^a^	Function	Fold difference	*P* value	FDR
MIXL1	Mix paired-like homeobox. Among its related pathways are Cardiac Progenitor differentiation and Adipogenesis.	142.7	3.52E–07	0.00142576
SLC30A3	Solute carrier family 30 (zinc transporter), member 3. Its related pathways are Transport of glucose and other sugars, bile salts and organic acids, metal ions and amine compounds and Transport of glucose and other sugars, bile salts and organic acids, metal ions and amine compounds.	23.8	5.32E–07	0.00142576
SFRP5	Secreted frizzled-related protein 5. Its related pathways are Wnt signaling pathway (KEGG) (Wnt proteins are secreted morphogens that are required for basic developmental processes) and Non-Canonical Wnt Pathway.	53.4	2.05E–06	0.00365475
AC007879.2	Uncharacterized	13.8	1.45E–05	0.00595947
AD000091.2	Uncharacterized	283.2	1.05E–05	0.00595947
C12orf44	Autophagy related 101	18.9	8.91E–06	0.00595947
HNF1A-AS1	HNF1A antisense RNA 1 affiliated with the antisense RNA class.	48.4	1.32E–05	0.00595947
LBR	Lamin B receptor. Its related pathways are Metabolism and Metabolism.	70.9	1.36E–05	0.00595947
MTMR1	Myotubularin related protein 1. Its related pathways are Metabolism and Metabolism.	20.4	1.44E–05	0.00595947
NLRP13	NLR family, pyrin domain containing 13	10.9	5.86E–06	0.00595947
PPP1R3G	Protein phosphatase 1, regulatory subunit 3G. It is related to protein phosphatase binding and glycogen binding*.*	82.1	1.25E–05	0.00595947
PRPSAP1	Phosphoribosyl pyrophosphate synthetase-associated protein 1. This gene is related to magnesium ion binding and enzyme inhibitor activity.	56.8	1.29E–05	0.00595947
RAB11FIP5	RAB11 family interacting protein 5 (class I). Its related pathways are Endocytosis and Delta508-CFTR traffic / ER-to-Golgi in CF.	53.4	1.01E–05	0.00595947
RP11-436F21.1	Uncharacterized	9.6	1.93E–05	0.00740049
C11orf95	Chromosome 11 open reading frame 95. Diseases associated with C11orf95 include ependymoma and chondroid lipoma.	119.4	2.56E–05	0.00913053
HOXD13	Homeobox D13. This gene is related to sequence-specific DNA binding transcription factor activity *and* chromatin binding.	96	3.07E–05	0.01028157
RP11-508M8.1	Uncharacterized	14.6	3.46E–05	0.0109017
CTC-504A5.1	Affiliated with the ncRNA class	9.9	3.82E–05	0.01135634
IRS4	Insulin receptor substrate 4. Its related pathways are Signaling by GPCR and Insulin receptor signaling cascade.	54.4	5.62E–05	0.01466991
MANEAL	Mannosidase, endo-alpha-like. It is related to *hydrolase activity*.	14.7	5.95E–05	0.01466991
MCU	Mitochondrial calcium uniporter. It is related to calcium channel activity and uniporter activity.	11.9	6.03E–05	0.01466991
SUV420H2_1	Uncharacterized	18.4	5.71E–05	0.01466991
SLC25A18	Solute carrier family 25 (glutamate carrier), member 18. It is related to this gene include symporter activity.	86.7	6.39E–05	0.01489091
GNAI1	Guanine nucleotide binding protein (G protein), alpha inhibiting activity polypeptide 1. Its related pathways are GPCR Pathway and GPCR Pathway.	10.8	6.69E–05	0.01492573
ADPRHL1	ADP-ribosylhydrolase like 1. It is related to magnesium ion binding and ADP-ribosylarginine hydrolase activity.	7.3	9.39E–05	0.01676156
CMTM4	CKLF-like MARVEL transmembrane domain containing 4. It is related to cytokine activity.	16	9.17E–05	0.01676156
F10	Coagulation factor X. it is related the pathways of Hemostasis and Formation of Fibrin Clot (Clotting Cascade).	11.4	8.76E–05	0.01676156
LPAR1	Lysophosphatidic acid receptor 1. Its related pathways are PI3K-Akt signaling pathway and Ras signaling pathway.	9.6	8.50E–05	0.01676156
LYSMD4	LysM, putative peptidoglycan-binding, domain containing 4	10.3	8.30E–05	0.01676156
RP11-886H22.1	Uncharacterized	9.3	9.38E–05	0.01676156
ATP12A	ATPase, H+/K+ transporting, nongastric, alpha polypeptide. It is related to the pathways of Ion channel transport and Transport of glucose and other sugars, bile salts and organic acids, metal ions and amine compounds.	10.2	1.22E–04	0.01943478
POU4F1-AS1	RNF219 antisense RNA 1	8.1	1.23E–04	0.01943478
PRDM13	PR domain containing 13. This gene related to methyltransferase activity.	30.5	1.23E–04	0.01943478
ZXDA	Zinc finger, X-linked, duplicated A. it is related to sequence-specific DNA binding transcription factor activity and C2H2 zinc finger domain binding.	45.3	1.17E–04	0.01943478
AC009495.4	Uncharacterized	8.9	1.59E–04	0.02368413
RP11–410N8.1	Uncharacterized	29.8	1.55E–04	0.02368413
ANKRD34B	Ankyrin repeat domain 34B	10.4	2.00E–04	0.02675171
DPY19L3	Dpy-19-like 3. It is related to transferase activity, transferring glycosyl groups.	5.2	2.08E–04	0.02675171
LINC00609	long intergenic non-protein coding RNA 609	8.9	2.08E–04	0.02675171
RP11–180P8.3	Uncharacterized	96.5	2.15E–04	0.02675171
RP11–492E3.2	Uncharacterized	−8.3	2.05E–04	0.02675171
TLX3	T-cell leukemia homeobox 3. Its related pathways are Transcriptional misregulation in cancer and SIDS Susceptibility Pathways.	83.1	1.96E–04	0.02675171
ZFHX3	Zinc finger homeobox 3. Its related pathways are Transcriptional Regulatory Network in Embryonic Stem Cell.	12.5	2.11E–04	0.02675171
SOHLH1	Spermatogenesis and oogenesis specific basic helix-loop-helix 1. It is related to sequence-specific DNA binding transcription factor activity and protein dimerization activity.	15.2	2.54E–04	0.03086759
SLC51A	Solute carrier family 51, alpha subunit. It is related the Bile secretion and Drug Induction of Bile Acid Pathway.	7.6	2.60E–04	0.03093234
TCF15	Transcription factor 15 (basic helix-loop-helix). It is related to ERK Signaling and ERK Signaling pathways.	8.8	2.74E–04	0.03191743
RP11–118B18.1	Uncharacterized	6.5	3.08E–04	0.03504717
ATP6V1G2	ATPase, H+ transporting, lysosomal 13 kDa, V1 subunit G2. A multisubunit enzyme that mediates acidification of intracellular compartments of eukaryotic cells.	23.3	3.68E–04	0.04059745
RP11–435M3.2	Uncharacterized	15.9	3.71E–04	0.04059745
C4orf32	A Protein Coding gene	4.4	4.10E–04	0.04218642
ZC3H8	Zinc finger CCCH-type containing 8. It is related to sequence-specific DNA binding transcription factor activity and sequence-specific DNA binding.	7	3.94E–04	0.04218642
ZXDB	A protein coding gene. It is related to nucleic acid binding.	32.1	4.03E–04	0.04218642
CHST10	Carbohydrate sulfotransferase 10. Its related pathways are Other types of O-glycan biosynthesis and Biological oxidations.	6.6	4.38E–04	0.04421968
RP11–7F17.5	Uncharacterized	−5.1	4.50E–04	0.04426996
TMEM52	Transmembrane protein 52	108.9	4.55E–04	0.04426996
NEURL	A Protein Coding gene. It is related the pathways of Signaling by GPCR and Disease.	6	4.68E–04	0.0447815
AP3B1	A protein coding gene. It is related to the pathway of Lysosome and Clathrin derived vesicle budding.	7.8	5.28E–04	0.04960697

^a^ *DEG* differentially expressed gene

Of the 56 DEGs in the HCV clearance group, 34 are known to encode biologically functional proteins. Of these 34 genes, 14 DEG gene products (25%) (MIXL1, SLC30A3, SFRP5, NLRP13, IRS4, GNAI1, CMTM4, F10, LPAR1, ATP12A, TLX3, ATP6V1G2, NEURL, AP3B1) are known to participate in, or to be related to, signal transduction pathways and innate immune responses, twelve DEG gene products (21%) (LBR, MTMR1, PPP1R3G, PRPSAP1, RAB11FIP5, MANEAL, MCU, ADPRHL1, PRDM13, DPY19L3, SLC51A, CHST10) are involved in metabolism and protein trafficking and eight DEG gene products (14%) (ZFHX3, SOHLH1, TCF15, ZC3H8, ZXDB, TLX3, HOXD13, ZXDA**)** are RNA/DNA binding proteins involved in gene regulation (Fig. 3b). However, no DEG was identified between plasma samples analyzed before and after HCV infection, regardless whether they were from clearance group (TR1B vs. TR1A) or chronic group (TR 2B vs. TR 2A) (Fig. 1).

**Fig. 4:**
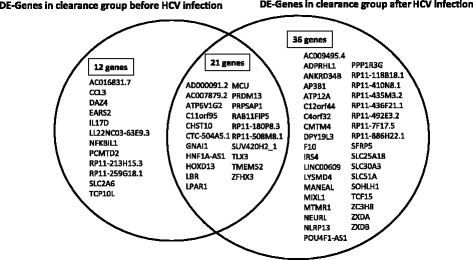
Intersectional analysis of the differentially expressed genes before and after HCV infection in clearance group and Chronic group. The diagram was generated with the statistical differentially expressed genes between clearance group and chronic group before and after HCV infection

**Fig. 5:**
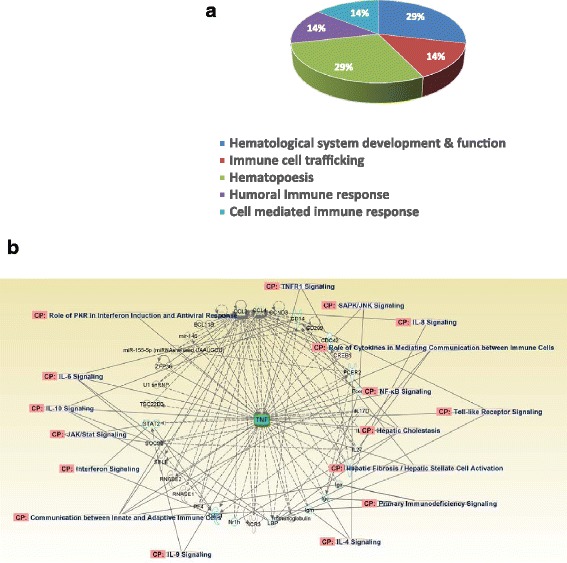
Physiological system development and function of unique DEG in clearance group before infection. **a** Pie chart demonstrates that majority of DEGs seen before HCV infection in the clearance group is clustered in immune mediated pathways. **b** Predicted interaction networks of mRNAs and associated pathways were identified using the DEGs in clearance compared to chronic group before HCV infection. The interactions between genes were identified using Ingenuity Pathway Analysis

Intersection analyses between DEGs identified in the clearance group before HCV infection (DEG-B, Fig. 1) and after HCV infection (DEG-A, Fig. 1) indicate that 12 DEGs were uniquely expressed in clearance group before HCV infection and 36 DEGs were uniquely expressed after HCV infection (Fig. 4). There were 21 DEGs that were common between the two groups. IPA analyses of these unique 12 genes expressed before HCV infection indicate that a number of these genes and their upstream regulators are associated with innate immune response in the clearance group (Fig. 5a and b). Among them 14% are involved in immune cell trafficking, 14% are involved in humoral immune response and 14% are involved in cell mediated immune response suggesting that most of the unique DEGs in the clearance group before infection may play a role in defending against the virus (Fig. 5a). For instance, cytokine (such as IL-1, IL-4, IL-6, IL-8, IL-9, IL-10, TNFR1, interferon) signaling, Toll like receptor (TLR) signaling, MAPK signaling, NF-κB signaling, and communication between innate and adaptive immune cells may be involved (Fig. 5b). Similar analyses with 36 DEGs after HCV infection found that these genes are involved in glucocorticoid receptor signaling, myc mediated apoptosis signaling, axonal guidance signaling, IGF1 signaling, MAPK signaling, p53 signaling, PI3K/AKT signaling, and acute phase response signaling.

## Discussion

Unraveling the mechanism of spontaneous HCV clearance after an acute phase of infection in HIV infected individuals provides valuable information that will be informative for the development of a vaccine against HCV. In a genome-wide association study of HCV infected individuals, SNPs in the region of the *IL28B* gene, encoding interferon-λ-3, were shown to be associated with the outcome of HCV infection; the CC genotype enhances spontaneous resolution of HCV infection, whereas those with the TT genotype showed a great propensity to develop chronic HCV infection [15, 16]. In the current study we could not detect any significant relationship between CC genotype and HCV clearance, because the CC genotype was found in four of the five clearance subjects and four of the eight chronically HCV infected subjects. The distribution of the genotypes in the infected subjects suggests that other factors beside the *IL28B* SNPs play a role in the spontaneous HCV clearance in the co-infected individuals [14, 18, 27]. However, the small sample size in our study also could explain our failure to detect an association between *IL28B* polymorphisms and HCV clearance.

Recent advances in RNA sequencing technology have provided opportunities to comprehensively and quantitatively sequence RNA and examine virus-host interactions at the transcriptional level. Two studies have reported transcriptomes analysis of liver tissues of HCV infected chimpanzees with spontaneous viral clearance or chronic infection outcomes. Nanda et al [18] reported that early induction of the genes associated with cell proliferation and immune activation, especially interleukin binding factor 3 (ILF3) and cytotoxic granule associated RNA binding protein (TIA1), was associated with subsequent HCV clearance in infected chimpanzees. Suet et al [28] reported that cellular genes induced by IFN-γ and those involved in antigen processing/presentation and the adaptive immune response were associated with HCV clearance. These reports suggest that host innate/adaptive immune responses play an important role in HCV clearance. However, studies on the transcriptome of liver tissues from HIV/HCV co-infected humans with different outcomes are very limited. Due to lack of available liver tissues, we focused on determining transcriptomes in plasma from HIV/HCV co-infected subjects with and without HCV clearance, since the quantity and characteristics of RNAs in plasma may reflect concurrent pathogenic changes in host liver tissues.

The availability of sequential plasma samples with defined onset and disease outcomes of HCV infection in highly characterized HIV-infected subjects from the MACS [25] provided us with a unique opportunity to identify, for the first time, the cellular transcripts that may be related to HCV clearance. Application of RNA sequencing technology in plasma has allowed us to determine such differentially expressed transcripts between subjects who cleared HCV and those who remained chronically infected after acute infection. In addition, the analysis of samples collected before and after acquisition of HCV infection provided information on cellular transcripts that may be associated with HCV clearance. Plasma transcriptome analysis of mRNA in subjects who cleared HCV and those who remained chronically HCV infected before and after HCV infection identified 12 DEGs that are uniquely expressed before HCV infection and a number of these genes and their upstream regulators are associated with innate immune response in the clearance group. For instance, IL17D, a member of IL17 family, plays a major regulatory role in host defense and inflammatory diseases [29]. The NFKBIL1 gene encodes a divergent member of the I-kappa-B family of proteins and is involved in the regulation of innate immune response by negatively regulating TLR and interferon regulatory factor (IRF) signaling pathways. In addition, the NFKBIL1 gene has a role in negative regulation of transcriptional activation of NF-kB genes in response to pro-inflammatory stimuli [30]. CCL3, a chemokine, has been shown to increase NK activity [31]. This goes along with observation that subjects who resolved HCV infection had a higher frequency of HCV-specific interferon-gamma-producing T-cells (*P =* 0.017) and cytotoxic NK-cells (*P =* 0.005), compared to patients who became chronically infected [27]. In addition, in HCV infected patients treated with interferon-α and ribavirin, increases in MIP-1α, MIP-1β and RANTES levels after 24 h of treatment were exclusively observed in the group that showed a sustained virological response, suggesting an antiviral role of CCL3 [32]. Furthermore, IPA analysis suggests that cytokine signaling (such as IL-1, IL-4, IL-6, IL-8, IL-9, IL-10, TNFR1, interferon), TLR signaling, MAPK signaling, NF-κB signaling, communication between innate and adaptive immune cells, and ELF3 could be involved in HCV clearance.

## Conclusions

Plasma RNA sequencing has identified the transcripts that are significantly associated with HCV clearance and are found to be expressed prior to infection. Some of the transcripts are involved in innate immune function. These results suggest that subjects who spontaneously clear HCV may have the transcripts that modulate forms of immunity that confer resistance to chronic infection with HCV. Further studies with longitudinal samples from HCV infected patients who cleared HCV infection, and those who became chronically infected may provide more definitive information about the nature of these inherited traits.

## Abbreviations

cDNA: Complementary DNA
DEG-A: Differentially expressed genes after HCV infection
DEG-B: Differentially expressed genes before HCV infection
DEGs: Differentially expressed genes
FDR: False Discovery Rate
HCV: Hepatitis C virus
HIV: Human immunodeficiency virus
IL28B: Polymorphisms in interleukin 28B
ILF3: Interleukin binding factor 3
MACS: The Multicenter AIDS Cohort Study
TLR: Toll like receptor
TR1A: HCV clearance group after HCV infection
TR1B: HCV clearance group before HCV infection
TR2A: Chronically HCV infected group after HCV infection
TR2B: Chronically HCV infected group before HCV infection
